# Dr. John Joseph Cassidy

**Published:** 1914-12

**Authors:** 


**Dr. John Joseph Cassidy,** Toronto 1869, died of heart disease at his home in Toronto August 1, aged 71. He was editor of the Canadian Journal of Medicine and Surgery for 16 years.

